# Effect of Functional Group and Carbon Chain Length on the Odor Detection Threshold of Aliphatic Compounds

**DOI:** 10.3390/s120404105

**Published:** 2012-03-27

**Authors:** Manuel Zarzo

**Affiliations:** Department of Applied Statistics, Universidad Politécnica de Valencia, Camino de Vera s/n, 46022 Valencia, Spain; E-Mail: mazarcas@eio.upv.es; Tel.: +34-963-877-490; Fax: +34-963-877-499

**Keywords:** olfaction, VOC, odorant, olfactometry, psychophysics

## Abstract

Odor detection thresholds (ODTs) are used for assessing outdoor and indoor air quality. They are obtained experimentally by olfactometry and psychophysical methods, and large compilations are available in the literature. A non-linear regression equation was fitted to describe the ODT variability of 114 aliphatic compounds based on the alkyl chain length for different homologous series (carboxylic acids, aldehydes, 2-ketones, esters, 1-alcohols, amines, thiols, thioethers and hydrocarbons). The resulting equation reveals an effect of the functional group, molecular size and also an interaction between both factors. Although the mechanistic interpretation of results is uncertain, the relatively high goodness-of-fit (R^2^ = 0.90) suggests that ODT values of aliphatic compounds can be predicted rather accurately, which is not the case for rigid molecules. This equation may serve as a basis for the development of more complex ODT models taking into account diverse structural features of odorants. The variability of power-law exponents was also investigated for the homologous series.

## 1. Introduction

The monitoring of volatile organic compounds (VOCs) is a key element in the assessment of outdoor and indoor air quality because many VOCs exert adverse impacts on human health and comfort. Health impact depends on the VOC toxicity and concentration. Odor detection thresholds (ODTs) are used to evaluate air quality with respect to human comfort. ODT is defined as the lowest concentration of a VOC than can be perceived or discerned from a blank stimulus or background noise. These thresholds are quantified by olfactometry and psychophysical methods with a panel of trained individuals [1,2]. Sensations at such low concentrations only reflect the experience of perceiving an indescribable smell significantly different from the blank. A higher concentration is required to recognize the odor character, which is called *odor recognition threshold*, but it is more difficult to measure reliably. ODTs are usually referred as concentration in air and expressed in ppm (v/v).

The relationship between perceived odor intensity and VOC concentration, which is known as psychophysical function or Stevens' law, depends on two parameters. Starting from a threshold concentration, odor intensity grows more or less quickly *versus* odorant concentration according to a parameter called *power-law exponent* [3,4]. This exponent is not equal for all VOCs, which implies that two odorants assessed at the same concentration may differ in their odor intensity even if their ODT is equal. Nonetheless, generally speaking, stronger odorants are detected at lower concentrations.

In odor psychophysics, the rated intensity of suprathreshold mixtures generally falls below the sum of intensities of the unmixed components. Olfactory mixtures can often lead to (1) masking or dominance by a stronger component [5], (2) averaging effect [6], (3) hypoadditivity [7,8], and (4) synergistic mixture interactions [9,10]. Inhibitory and synergistic effects seem to be related to the power-law exponents of odorants in the mixture [11].

The potential of a particular VOC to cause odor problems depends on its ODT and volatility. For example, methanethiol and 1-dodecanethiol present a similar ODT but their vapor pressure is remarkably different (1,300 and 0.009 mm Hg at 25 °C, respectively). Consequently, the odor pollution potential or odorant *potency* is best described by the quotient of its headspace concentration at saturation and its odor threshold concentration, which is called *odor index* [12]. A similar concept is the *odor value*, also called *odor unit number*, which is defined as the quotient of the actual concentration of a given odorant and its threshold concentration [4]. In olfactory mixtures, the sum of odor units for all components is correlated with the perceived smell intensity of the mixture [13].

Van Gemert has gathered almost 18,000 threshold values from about 3,000 references, which is currently the most comprehensive ODT compilation [14]. Fazzalari's database contains odor and taste thresholds on 2,626 chemicals [15]. Devos and coworkers published standardized ODTs for 529 compounds [16]. Other compilations with much smaller numbers of VOCs are also available [12,17,18].

Olfactory thresholds may be remarkably different even for two VOCs with a similar molecular structure. Although some authors have attempted to predict ODT on a mechanistic basis for a set of compounds from different homologous series [19,20], the molecular characteristics affecting odor detectability still remain basically unknown. This is not surprising given that about 350 functional human olfactory receptors are involved in odorant recognition [21].

Many works have proven that very similar molecules may have rather different ODT values [22,23], even in the case of enantiomers [24,25]. However, most pairs of strong-weak odorants with a similar structure are rather rigid molecules due to the presence of cyclic structures, aromatic rings or double bonds that reduce the molecular flexibility. To what extent does ODT vary for compounds belonging to the same homologous series, *i.e.*, for VOCs with the same functional group but varying in the alkyl chain length? Is the effect of carbon chain length on ODT similar for different homologous series? Few studies have tackled this issue [26] due to the limitations of obtaining experimental ODTs for a large set of compounds. The main target of the present study was to evaluate the ODT variability according to functional group and alkyl chain length for 114 compounds with aliphatic substituents. The variability of power-law exponents from the literature [3] was also investigated.

## 2. Experimental Section

ODTs reported by different laboratories can be extremely different, which becomes a serious problem when comparing olfactory thresholds from literature sources. Much of the variation among laboratories seems to be attributable to issues of experimental precision, especially concerning control of VOC concentration [27]. Devos and coworkers [16] applied an algorithmic procedure to ODTs compiled from the literature in order to correct the bias and increase their accuracy, resulting a comprehensive inventory of corrected thresholds for 529 compounds. Although ODT is usually expressed in ppm, this database uses logarithmic units (pOL) according to Equation (1). Equation (2) indicates the conversion from pOL to ppm units. Molar concentrations can be derived from pOL units according to Equation (3):

(1) $$\text{pOL}=-\log_{10}[10^{-6}\cdot \text{ODT}(\text{ppm})]$$

(2) $$\text{ODT}[\text{ppm}(\text{v}/\text{v})]=10^{\left(6-\text{pOL}\right)}$$

(3) $$\text{ODT}(\text{mol}/\text{L})=10^{-\text{pOL}}/24$$

From this database, I selected all VOCs with a saturated aliphatic chain (*i.e.*, without cycles, substituents or halogens) and containing a single functional group: carboxylic acids, aldehydes, 2-ketones, esters, 1-alcohols, amines, thiols and (mono/di/tri)thioethers. Alkyl unbranched hydrocarbons were also considered. The resulting set of 114 compounds (The set of 114 compounds and their pOL values (obtained from [16]) are available as Supplementary Material) contains valuable information to study the effect of functional group and carbon chain length on ODT. The variability of power-law exponents, obtained from Devos' compilation [3], was also studied for these homologous series.

## 3. Results and Discussion

**Figure 1.**
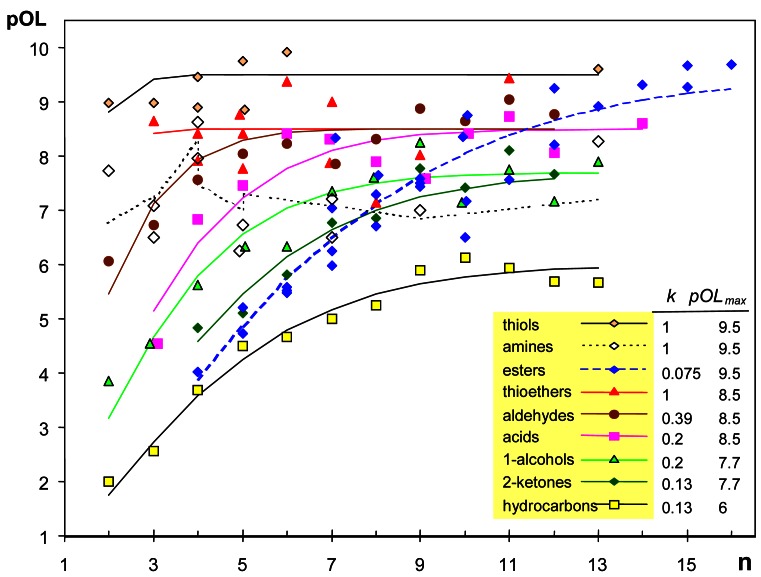
Plot of pOL *versus* **n** (number of atoms except hydrogens) for 114 aliphatic compounds. Equations in Table 1 have been fitted for each homologous series. Estimated values of *pOL_max_* and *k* (coefficients of Equation (4)) are indicated within the graph.

A scatterplot of pOL *versus* **n** (number of atoms except hydrogens) shows a positive correlation (Figure 1). More precisely, pOL tends to an asymptotic value that depends on the homologous series. Multiple linear regression does not deal properly with asymptotic trends, and a non-linear regression Equation (4) was fitted using the software Statgraphics 5.1. This equation has two coefficients (*k* and *pOL_max_*), being **n** the independent variable:

(4) $$\text{pOL}=[1-\exp(-k{\text{n}}^{1.4})]\cdot {\text{pOL}}_{\text{max}}$$

Fitted curves for the different homologous series are not parallel, which implies an interaction between alkyl chain length and functional group. The coefficient *pOL_max_* is the maximum pOL reached asymptotically as **n** increases, while *k* is related to the initial slope of the curve. The exponent of **n** was set after trying different values from 1.0 to 2, and 1.4 was the value that achieved the best goodness-of-fit for all homologous series.

It was found that pOL values of amines are better fitted by adding the term (8.6-pK_a_) to Equation (4) (see Table 1), being pK_a_ the acid dissociation constant. The effect of pK_a_ is related to the affinity to react with water, and suggests that the uncharged form of amines is the one responsible for their low olfactory threshold and strong smell. Actually, if pK_a_ increases one unit, the concentration of the uncharged form is ten-fold decreased and pOL is reduced in one unit according to the proposed predictive equation for amines.

Two outliers (methyl butanoate, pOL = 8.33 and hexyl acetate, pOL = 6.5) were identified by plotting the residuals of the fitted model on a normal probability plot, and they were discarded. The estimated values of regression coefficients are shown in Figure 1 and Table 1, resulting a coefficient of determination R^2^ = 0.90.

Figure 1 reveals a clear effect of the functional group. Chemists noticed long ago that the presence of certain chemical groups in a molecule is frequently correlated with a particular odor [28]. Reported evidence has shown that organic chemists were reasonably successful in identifying the functional groups of unfamiliar odorants [29]. Results reported here highlight that the effect of functional group is apparent not only in odor character but also in odor detectability.

The lowest pOL values correspond to hydrocarbons, probably due to their lower solubility and diffusivity in the mucus layer of the olfactory epithelium. The highest *k* values correspond to thiols, thioethers and amines, which implies that short molecules of these types are detected at lower concentrations than 1-alcohols, 2-ketones or ethers. Although alcohols (-OH) and thiols (-SH) are similar functional groups, it is well known by chemists that thiols are likely to be detected at lower concentrations and to smell strongly. Actually, thiols present the highest *pOL_max_* and *k* values, resulting a fitted curve which is nearly horizontal.

The observed effect of functional group is difficult to interpret, as well as the fact that pOL grows asymptotically as the carbon chain length increases. The ODT of small esters and 2-ketones is similar, which is intuitively appealing because both contain a carbonyl moiety. However, large esters tend to a *pOL_max_* similar to thiols. Although different hypotheses proposed in the literature can explain some of these effects (see [21] for review), the mechanistic interpretation of ODT variability is still rather uncertain due to the multi-step processes involved in olfaction: odorant diffusion in the mucus layer, effect of odorant-binding proteins, biotransformation enzymes, and interaction with a large repertoire of olfactory receptors [21].

Saito and coworkers [30] have tested the responses of 219 mouse olfactory receptors (ORs) and 245 human ORs (which accounts for 63% of human OR genes) to a panel of 93 odorants. Dose-response curves were obtained for every combination of the 129 receptors and 67 odorants that showed a response. Data were fitted to a sigmoidal curve and EC50 values were obtained (*i.e.*, the concentration for which half of the response was achieved). It was found that 1-octanol and octanethiol activated five ORs. Similarly, 1-nonanol and nonanethiol activated four and seven receptors, respectively. Although one particular OR responded to both thiols but not to any other tested odorant, which might explain their sulfur smell, similar EC_50_ values were obtained for thiols and their homologous alcohols. Thus, neither the number of ORs activated by thiols nor the affinity to bind their target ORs would explain why thiols are detected at a concentration which is several orders of magnitude lower than their homologous alcohols. One hypothesis would be that thiols might act as competitive inhibitors of cytochrome P-450 enzymes present in the olfactory epithelium [21]. These enzymes are involved in reactions of degradation and biotransformation that inactivate and clear up the dissolved VOCs once they are perceived.

The work of Devos *et al.* [3] provides corrected power-law exponents for 213 odorants, 95% of which range from 0.23 to 0.76, with a mean value of 0.44 and a median of 0.40. It turns out that these exponents are negatively correlated with **n** (number of atoms except hydrogens) (*r* = −0.26, *p* = 0.0001). If a quadratic model is fitted, the resulting equation suggests that power-law exponents (PLE) decrease asymptotically as **n** increases. Based on this observation, a nonlinear regression Equation (5) was fitted. According to this model, an exponent of 0.39 is expected for large molecules. The goodness-of-fit is low (R^2^ = 0.094) in part due to the uncertainty of PLE values, most of which (60%) were obtained from a single literature source [3]:

(5) PLE=0.386+0.36⋅exp(−0.3n)

Interestingly, the five highest power-law exponents correspond to ammonia (1.08) and amines, which reveals an effect of the functional group as in the case of ODT. Thus, the nonlinear model *a* + *b*·exp(−0.3 **n**) was fitted for the different homologous series of aliphatic compounds. Molecules with double bonds and methyl substituents were also considered as it seems that these structural features do not affect PLE. The resulting equations (Table 1) suggest that higher power-law exponents correspond to small molecules with certain functional groups such as aldehydes and alcohols (Table 1).

If power-law exponents from [3] are compared with pOL values from [16], it turns out that both parameters are negatively correlated (*r* = −0.43, *p* < 0.0001). This correlation, which was discussed by other authors [31], is expressed by Equation (6) being I_N_ an indicator variable that takes the value 1 for compounds containing nitrogen and zero otherwise. The coefficient of determination is R^2^ = 0.33. Figure 1 reflects an effect of functional group in pOL values that is more apparent for small molecules (thiols > aldehydes > 1-alcohols > 2-ketones). The opposite trend is observed for power-law exponents as reflected by equations in Table 1, which is consistent with the observed negative correlation between pOL and PLE:

(6) $$\text{PLE}=0.738-0.0425\cdot \text{pOL}+0.18\cdot {\text{I}}_{\text{N}}$$

Previous studies have attempted to predict power-law exponents based on physicochemical parameters such as molecular heat capacity [32], parachor [33], molar volume at boiling point, ability to accept hydrogen bonding, or electronic polarizability [34]. Wright [31] has suggested that relatively high PLE values are associated with relatively inflexible molecular structures, which is true for compounds such as cyclohexanol (0.65), cyclopentanone (0.61) or toluene (0.51). However, other similar molecules present low exponents such as phenol (0.27), benzenethiol (0.24) or cyclohexene (0.30). Thus, the effect of molecular rigidity is uncertain, and larger compilations of PLE values would be necessary to further investigate their variability based on molecular descriptors and physicochemical parameters.

## 4. Conclusions

Although the interpretation of results is quite uncertain, the goodness-of-fit achieved by the proposed ODT model (R^2^ = 0.90) indicates that this parameter can be predicted rather accurately for homologous series of aliphatic compounds. Equation (4) may serve as a framework to develop more complex and comprehensive predictive ODT models taking into account diverse structural features of odorants. Consistent with previous studies [32–34], results reported here also suggest that power-law exponents are related to molecular structure, particularly for small molecules. The development of predictive equations for ODT and PLE will provide a comprehensive framework to characterize the relationships of odor strength according to molecular structure.

Further studies are encouraged to better understand correlations between odor thresholds and structural parameters with respect to the type, number and position of functional groups, length of alkyl chains, and number and position of branching points [26].

## Figures and Tables

**Table 1.** Fitted equations for ODT (data from [16]) and power-law exponents (data from [3]) corresponding to different homologous series (“Num” stands for the number of compounds used in each ODT equation).

**Compounds**	**Num**	**Equations for ODT (in pOL units)**	**Equations for power-law exponents (PLE)**
Thiols	8	pOL = 9.5 · [1 − exp(−**n**^1.4^)]	PLE = 0.36
Amines	11	pOL = 9.5 · [1 − exp(−**n**^1.4^)] *+* (8.6 − pK_a_)	PLE = 0.39 + 1.15·exp(−0.3 **n**)
Esters	26	pOL = 9.5 · [1 − exp(−0.075 **n**^1.4^)]	PLE = 0.36 + 0.33·exp(−0.3 **n**)
Thioethers	12	pOL = 8.5 · [1 − exp(−**n**^1.4^)]	PLE = 0.36
Aldehydes	11	pOL = 8.5 · [1 − exp(−0.39 **n**^1.4^)]	PLE = 0.36 + 0.33·exp(−0.3 **n**)
Acids	11	pOL = 8.5 · [1 − exp(−0.2 **n**^1.4^)]	PLE = 0.36
1-Alcohols	12	pOL = 7.7 · [1 − exp(−0.2 **n**^1.4^)]	PLE = 0.36 + 0.51·exp(−0.3 **n**)
2-Ketones	9	pOL = 7.7 · [1 − exp(−0.13 **n**^1.4^)]	PLE = 0.39 + 1.15·exp(−0.3 **n**)
Hydrocarbons	12	pOL = 6.0 · [1 − exp(−0.13 **n**^1.4^)]	PLE = 0.39
